# Development and validation of the Teen Moms Child Feeding Questionnaire for Sub-Saharan Africa

**DOI:** 10.1186/s12889-023-16365-5

**Published:** 2023-08-04

**Authors:** Mercy E. Sosanya, Isaiah Beamon, Raza Muhammad, Jeanne H. Freeland-Graves

**Affiliations:** 1 Department of Nutritional Sciences, University of Texas at Austin, Austin, USA; 2 Department of Nutrition and Dietetics, The Federal Polytechnic, Bauchi, Bauchi, Nigeria

**Keywords:** Knowledge, Attitudes, Breastfeeding, Complementary feeding, Infant and young child feeding, Adolescent mother, Psychometric properties, Sub-Saharan Africa

## Abstract

**Background:**

In Sub-Saharan Africa, the nutritional status of children born to teenage mothers deserves critical attention. Maternal knowledge and attitudes concerning infant and young child feeding (IYCF) may predict actual practices and child nutritional status. This study created and validated the Teen Moms Child Feeding Questionnaire for Sub-Saharan Africa.

**Methods:**

A literature search on IYCF knowledge and attitude gaps in teenage mothers generated scale items. Ten nutrition experts and six teenage mothers assessed content validity and comprehensibility, respectively. Construct validation was conducted by item response theory (IRT) and confirmatory factor analysis (CFA), in 150 teenage mothers in rural communities of Abuja, Nigeria. Model fit parameters were estimated by standardized chi-square tests. Internal consistency reliability was determined by marginal reliability and Cronbach’s alpha. In a sub-sample of 40 women who completed the questionnaire two weeks later, test–retest reliability was assessed via intraclass correlations.

**Results:**

The IRT analysis retained 23 knowledge items on infant food type, breastfeeding and complementary feeding, with acceptable discrimination and difficulty. CFA produced a six-factor solution (exclusive breastfeeding, breast milk expression, meal frequency, responsive feeding, dietary diversity, and barriers) with 17 attitude items. Confirmatory fit and Tucker Lewis indices > 0.9; Root Mean Square Errors of Approximation and Standardized Root Mean Square Residuals < 0.08, showed good model fit. Overall Cronbach’s alpha of the attitude scale (0.843), subscales (≥ 0.6) and high intraclass correlation coefficients (> 0.75) indicated reliability.

**Conclusion:**

The Teen Moms Child Feeding Questionnaire for Sub-Saharan Africa is a valid assessment tool for IYCF knowledge and attitudes of teenage mothers.

**Supplementary Information:**

The online version contains supplementary material available at 10.1186/s12889-023-16365-5.

## Background

Infant and young child feeding (IYCF) practices are pivotal determinants of the nutritional status, development, health and survival of children within the first 1000 days of life [1]. The World Health Organization (WHO) and the United Nations Children’s Fund (UNICEF) recommend that neonates be breastfed within one hour of birth (early initiation); receive breastmilk alone from 0 – 6 months (exclusive breastfeeding); and continue to be breastfed until 24 months (continued breastfeeding); coupled with appropriate complementary feeding [2]. Breastfeeding practices that fail to meet these recommendations are defined as sub-optimal [3, 4]. For complementary feeding, optimal recommendations are the timely provision of safe, age-appropriate foods, fed responsively in appropriate amounts, and with adequate diversity and frequency [1, 5]. Adequate dietary diversity is described as the provision of foods from a minimum of five out of eight distinct food groups including breast milk, for children 6 – 23 months [1]. To achieve minimum meal frequency, children 6 – 8 months should receive complementary foods at least 2—3 times daily; and 3 – 4 times from 9 – 23 months, along with healthy snacks 1 – 2 times/day [6].

In Sub-Saharan Africa, over 63 million annual cases of morbidity associated with diarrhea, respiratory illnesses, and childhood obesity, as well as over 300,000 deaths in children 0-23 months, are attributable to lack of being breastfed [7]. Furthermore, lack of breastfeeding has been estimated to result in substantial economic losses of up to $42 billion annually in the region, through avoidable health care costs, mortality, and cognitive losses [7]. A study in Nigeria showed that children born to mothers who initiated breastfeeding more than one hour after delivery rather than immediately, had a ten-fold likelihood of stunting (low height-for-age), and a seven-fold likelihood of being underweight [8, 9]. Those that were breastfed for less than six months were twice more likely to be wasted (low weight-for-height) [8, 9]. A 2017 meta-analysis of the impact of interventions to improve complementary feeding has shown small, but significant effects on both linear and ponderal growth [10]. But more recent findings indicate reductions in 17 – 21% of stunting associated with adequate complementary feeding [11, 12]. In Sub-Saharan Africa, the nutritional status of children born to teenage mothers deserves critical attention, because 32 – 74% of adolescent girls (≤ 19 years) living in 21 countries have begun childbearing [13]. According to a study in Ghana, children born to adolescent mothers had a thirteen-, eight- and three-fold likelihood of underweight, stunting, and wasting, respectively, and were over 50% more likely to die within the neonatal period, as compared to children of older mothers [14].

Previous studies among teenage mothers suggest that maternal education, knowledge, and attitudes towards child feeding can influence IYCF practices [15–20]. A study conducted in Bangladesh identified major gaps in the IYCF knowledge of adolescent mothers. These include lack of awareness of: the appropriate time to initiate breastfeeding; the need to avoid prelacteal feeds and other fluids/foods for the first six months; responsive feeding; and importance of a diverse diet [21]. A recent review of qualitative studies has shown that the IYCF knowledge of adolescent mothers was more limited in comparison to older mothers, and that these teenage mothers had inadequate knowledge concerning how frequently babies need to be breastfed, or of signs that the infant is receiving sufficient breast milk [22]. Reports have indicated that knowledge of mothers concerning breastfeeding and complementary feeding is predictive of actual child feeding practices and is associated with height-for-age and weight-for-height z-scores in children [23, 24]. Similarly, higher prenatal attitude scores and breastfeeding self-efficacy have been associated with appropriate practices concerning breastfeeding initiation, duration and continuation [22]. Another study in Ghana showed that a positive attitude may not always translate to adequate IYCF practices [18]. Nonetheless, an integrative review has shown that prenatal and post-partum attitudes towards breastfeeding influenced breastfeeding choices of adolescent mothers [25].

Instruments to measure both knowledge and attitudes of teenage mothers towards child feeding are essential to provide empirical data for evaluating current status, and for devising strategies to improve child feeding practices in this population. Since 2014, several questionnaires have been utilized for assessment of IYCF indicators [1, 26–30]. Other tools exist for measurement of breastfeeding intention and nutritional knowledge of women of child-bearing age [31–34]. Nonetheless, the existing questionnaires were either designed for generic maternal populations regardless of age [26, 35]; focused on other populations (unmarried adolescents, young adults, health workers or student nurses) [28, 29, 31, 32]; solely measured factors related to breastfeeding [30, 31]; or addressed child feeding practices alone, and not maternal knowledge and attitudes [34]. Yet, to the best of our knowledge, there is presently no instrument for evaluating the knowledge and attitudes of mothers concerning breastfeeding and complementary feeding, specifically validated in teenage mothers in Sub-Saharan Africa. Thus, this study was designed to create and validate a Teen Moms Child Feeding Questionnaire for use in Sub-Saharan Africa.

## Methods

### Development of the Teen Moms Child Feeding Questionnaire for Sub-Saharan Africa

**Fig. 1:**
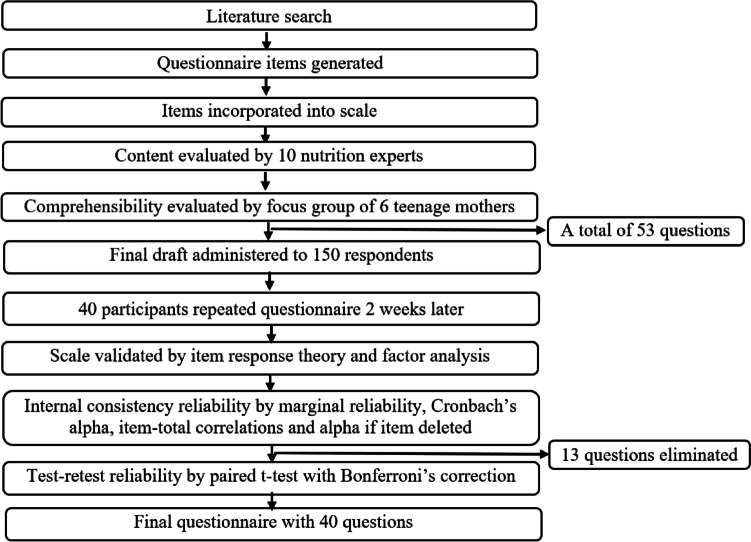
Flowchart of development and validation of the Teen Moms Child Feeding Questionnaire for Sub-Saharan Africa

The process workflow for development of the Teen Moms Child Feeding Questionnaire is shown in Fig. 1. Initially, an extensive review of existing literature was conducted to identify reported gaps in the IYCF knowledge and attitudes of teenage mothers. The review of literature focused on breastfeeding, complementary feeding, and existing questionnaires, including the WHO IYCF questionnaire and the Food and Agriculture Organization (FAO) model questionnaires for assessing nutrition-related knowledge, attitudes, and practices [1, 26]. Items were generated to create a preliminary instrument and incorporated into a scale to measure IYCF knowledge and attitudes of teenage mothers. Three constructs (perceived benefits, perceived barriers and self-efficacy) from the Health Belief Model were employed in generating items for the attitude scale [36]. The items were created for interviewer administration due to low literacy levels among the study population [37, 38]. Evaluation of this initial instrument for content validity, ease of readability and relevance was conducted by a panel of 10 nutrition experts pooled from the Department of Nutritional Sciences at the University of Texas at Austin; Department of Nutrition and Dietetics at the Federal Polytechnic, Bauchi, Nigeria; Department of Human Nutrition and Dietetics, University of Ibadan, Nigeria; and the Department of Family Health at the Federal Ministry of Health, Abuja, Nigeria. After scale revision, a focus group of six teenage mothers living in rural areas of Abuja, Nigeria, discussed the comprehensibility of the questionnaire. The final version of the questionnaire incorporated qualitative feedback from the expert panel and focus group. As English is the official language in Nigeria and Hausa is the most common language in the study population, questionnaires were created in English, translated by trained interviewers into the Hausa language, and participant responses were back translated from Hausa to English. Questionnaires were completed using paper-and-pencil.

### Demographic survey

A demographic questionnaire was created based on the FAO model questionnaires for nutrition-related knowledge. The survey collected information on the age of the mother, education, location of residence, marital status, living situation, employment status, personal and household income, household size and parity, as well as the age of the index child, birth order and gender.

### Study design and participants

A cross-sectional study was conducted to explore the psychometric properties of the Teen Moms Child Feeding Questionnaire. Adolescent mothers (ages 14 – 19 years, *n* = 150), with at least one child younger than two years old, were recruited from the rural suburbs of Abuja, Nigeria. With over 211 million inhabitants, Nigeria is a country with a diverse population comprised of over 250 ethnic groups. Nigeria has the largest population in Africa and is home to one of every six persons living in Sub-Saharan Africa [39, 40]. The 2021 Human Development Index (HDI) of Nigeria (0.54) is almost equivalent to the HDI for Sub-Saharan Africa as a whole (0.55) [41, 42]. Approximately 60% of adolescent girls living in the rural areas of Nigeria have borne a child [13]. Thus, this country was selected for this research.

Recruitment was done in-person, from March to July 2022, in central locations of Angwan-Sayawa, Dape, Gosa, Iddo-Pada, Kabusa, Karmo, Kagini and Sabon-Lugbe communities of Abuja Area Municipal Council, Abuja, Nigeria. Community access was provided by the Center for Family Health Initiative, a non-profit organization working in the area. The study was publicized through community leaders and local communication channels. All interested teenage mothers were interviewed to ascertain eligibility for the study. Research personnel explained the study procedures, risks, and benefits to potential participants, in English and Hausa. Written, informed consent was obtained from eligible teenage mothers who chose to participate in the study. Since some of the mothers were minors, consent via signature was also obtained from their adult husbands or parents. Participants completed both the demographic survey and the Teen Moms Child Feeding Questionnaire in one visit (first time-point) and were compensated in cash (~ 2,265 Naira or $5) [43]. The research team reiterated instructions and reminded participants to provide answers based on what they believed to be correct, and not just what they thought was socially desirable.

Contact details were obtained for participants who expressed interest in completing the questionnaire at a second time point. A sub-sample of 40 mothers completed the instrument two weeks later, for test–retest reliability. All questionnaires and informed consent forms were de-identified and coded as subject numbers on the data sheets and questionnaires. These were then stored in a locked cabinet in a locked office for confidentiality.

### Teen Moms Child Feeding Questionnaire for Sub-Saharan Africa

The knowledge domain of the Teen Moms Child Feeding Questionnaire initially contained 31 items that tested maternal IYCF knowledge on food type for an infant, exclusive breastfeeding, and complementary feeding. Questions were either open-ended, or multiple choice with four answer choices and only one correct option. To ensure that the data were captured correctly, all responses to the open-ended questions were reviewed, and preliminary dichotomous codes of “correct” and “incorrect” were generated [44]. Each response was then reviewed and placed in either of these categories, depending on whether or not they reflected knowledge of the concepts being tested. Incorrect responses to the items were scored as 0, and each correct response was scored as 1. All the responses were coded a second time, and confirmed a third time, to minimize errors [45].

Psychometric evaluation via Item Response Theory [46] resulted in retention of 23 questions (8 questions deemed as unusable). Each correct score was worth 4 points, for a total possible score of 92 points from 23 items. Item scores were then summed to yield a composite score for each of the three sub-categories of knowledge topics. Composite scores were computed from three items for infant food type, twelve items for knowledge of exclusive breastfeeding sub-section, and eight items for complementary feeding. Respondents with scores below the mean were rated as having inadequate knowledge; those scoring between the mean and 1 standard deviation were considered as having average knowledge; and scores above 1 standard deviation were rated as having adequate IYCF knowledge.

The attitude section initially contained 23 items that measured perceived benefits, perceived barriers and self-efficacy concerning exclusive breastfeeding, breast milk expression, frequent complementary feeding, dietary diversity, and responsive feeding. Item responses were on a 5-point Likert scale from strongly disagree to strongly agree. For ease of interpretation, “strongly disagree” and “disagree” were coded as -1; “neither disagree nor agree” as 0; and “agree” and “strongly agree” as + 1. Confirmatory factor analysis (CFA) produced a final attitude scale containing 6 subscales and 17 questions. All negatively worded items on the attitudes section of the questionnaire were reversely worded and then scored. The range of possible scores for each of the first five subscales was from – 3 to 0 to + 3, since each of these had three items. The range of possible scores for the last subscale was from – 2 to 0 to + 2, since it had two items. Item scores for each subscale were summed up for each participant. Participants with negative total scores on each subscale were rated as having a negative attitude towards the respective child feeding indicator, and vice versa. Participants with a zero total score were rated as indifferent. The final questionnaire contained a total of 40 questions.

### Statistical analyses

Data were analyzed using R software version 4.2.2 and the R studio environment version 2022.07.2 + 554 [47]. Descriptive statistics including frequencies, percentages, means, standard deviations and median were computed for demographic variables.

### Validity

#### Construct validity of the knowledge scale

The dichotomously scored knowledge section of the Teen Moms Child Feeding Questionnaire was psychometrically evaluated using the Two-Parameter Logistic (2-PL) Item Response Theory (IRT) [48]. Item discrimination levels from 0.35 to 2.5 were regarded as acceptable [46, 49], and used as criteria for question retention. Acceptable difficulty levels can range from -3 (very easy) to 0 (average difficulty) to 3 (very hard), and question items within this range were retained [46, 50]. The IRT models have been utilized in psychometric analyses to calibrate a question bank to measure the influence of parental practices on child dietary habits [51]. Also, these models have been applied to the development and refinement of diet quality scales for school children in Brazil, as well as in the validation of a tool to measure nutritional knowledge in European adolescents [48, 52].

Unidimensionality of the knowledge scale was ascertained by a modified iteration of Horn’s parallel analysis (modified parallel analysis plot in the supplementary material) [53, 54]. The model fit of the knowledge scale was determined via the Cochran-Mantel–Haenszel test, commonly represented as M_2_ [55, 56]. Item goodness-of-fit was assessed by standardized chi-square (*S-X*2), using a Monte Carlo simulation [57]. Scale items with p-values greater than 0.05 reflected a good fit with the hypothetical model [58].

#### Construct validity of the attitude scale

The factorability of the attitude scale was determined using Bartlett’s test of sphericity, along with the Kaiser–Meyer–Olkin (KMO) measure of sampling adequacy [59]. Latent constructs in the attitude domain of the Teen Moms Child Feeding Questionnaire were identified through exploratory factor analysis (EFA), using generalized least squares estimation and oblimin rotation with assumed correlations of the underlying constructs [60]. Determination of the number of factors to extract was based on having eigenvalues greater than 1.0 [61]. Items with factor loadings ≥ 0.3 were regarded as acceptable, because this indicates at least a moderate correlation between the item and the factor [62–64]. The factor pattern obtained from the EFA was verified by CFA [65].

Chi-square goodness-of-fit test was used to evaluate the model fit of the attitude scale [66]. The chi-square model produces other goodness-of-fit indices, including Tucker Lewis Index (TLI), Comparative Fit Index (CFI), root mean square error of approximation (RMSEA) and standardized root mean square residual (SRMR). These indices also were computed to evaluate the fit of both the knowledge and attitude scales [67]. Values > 0.9 for both the CFI and TLI reflected good model fit [68, 69]. The RMSEA and SRMR values less than 0.08 were regarded as acceptable [70–72].

The chi-square is reported routinely in results of factor analyses because it is the original fit index and the foundation for other indices [73]. Yet, the use of this test has numerous limitations including sensitivity to sample size, model complexity, violations of the assumption of normality, and missing variables [70, 73, 74]. Due to these limitations, other absolute indices are often reported in goodness-of-fit analyses, including the goodness-of-fit index, adjusted goodness-of-fit index and normed chi-square [70, 73, 74]. The model in the present study was relatively complex, consisting of 16 variables. Thus, the normed chi-square (chi-square divided by the degrees of freedom or χ2/df) was reported in addition to the chi-square values. In the absence of absolute standards for the normed chi-square, ratios of χ2/df of 2 and 3 represent "good" or "acceptable fit, respectively [70, 74].

### Reliability

#### Reliability of the knowledge scale

The internal consistency of the knowledge scale was confirmed by marginal reliability. Marginal reliability is one of two measures of reliability often employed in IRT analyses [75]. It expresses the relationship between the true score and total variances, as a function of the estimated latent trait [76]. This method has been utilized in reliability assessment of a child feeding knowledge scale for childcare providers [49]. The marginal reliability index can take any value from 0.0 to 1.0, with values closer to 1.0 considered good [45].

#### Reliability of the attitude scale

Cronbach’s coefficient alpha was computed to determine the internal consistency reliability for the attitude scale, and all its identified sub-scales [75]. A scale with a Cronbach’s alpha between 0.6 and 0.7 is regarded as acceptable, and a value of 0.8 or higher indicates a high level of reliability [77]. To further ascertain reliability, the Cronbach’s alpha if item deleted was computed [78]. Further confirmation of the reliability of the attitude scale was determined by the item-total correlations, which measure the consistency between each item and the others in a scale [79]. A large item-total correlation establishes that the construct being measured by a specific item is the same with other items in the model, and the suggested minimum value is 0.3 [79, 80].

To determine test–retest reliability, estimates of intraclass correlation coefficients with 95% confidence intervals were computed based on a single-rater, absolute agreement, two-way mixed effects model, using measurements collected from a sub-sample of respondents who completed the instrument two weeks later [81, 82]. Intraclass correlation coefficients < 0.4 are considered poor, 0.4 — 0.59 regarded as fair, 0.6 — 0.74 deemed good and estimates > 0.75 accepted as excellent [82, 83].

### Ethical considerations

This study received ethical approval from the Institutional Review Board of the University of Texas at Austin (ID number STUDY00001047), and the Health Research Ethics Committee of the Federal Capital Territory, Abuja, Nigeria (approval number FHREC/2021/01/148/14–12-21).

## Results

Table 1 summarizes the demographic characteristics of the 150 participants who completed the Teen Moms Child Feeding Questionnaire for Sub-Saharan Africa. A large percentage of the mothers (*n* = 132, 88%) were over 17 years, had attended secondary school (*n* = 113, 75.3%), were married (*n* = 83, 55.3%), and living with husbands (*n* = 77, 51.3%). Similarly, majority (over 70%) of the mothers were not working and had no income. About one-fifth (*n* = 33, 22%) of these teenage mothers could not estimate household monthly incomes. Typical monthly household incomes were ≤ 20,000 Naira (≤ 44 USD), with a median of 15,000 Naira (33 USD). Two-fifths of the households of respondents (*n* = 60) were within this income bracket. More than half (53.2%) of the index children were male, with about one-third (32.7%) within the ages of 0 – 5 months.

**Table 1:** Socio-demographic characteristics of teenage mothers in rural areas of Abuja, Nigeria (*n* = 150)

Characteristics	n	%
**Mother**		
**Age, years**		
15–17	18	12.0
18 – 19	132	88.0
**Education**		
None	4	2.7
Primary	28	18.7
Secondary	113	75.3
Tertiary	5	3.3
**Marital status**		
Single	67	44.7
Married	83	55.3
**Living with**		
Parents, relatives	73	48.7
Husband	77	51.3
**Occupation**		
None	106	70.7
Services (hairdressing, housekeeping, tailoring)	32	21.3
Other	12	8.0
**Personal monthly income (Naira, USD)**		
None	105	70.7
**≤ **20,000 Naira (**≤ **USD 44)	40	26.7
> 20,000 Naira (> USD 44)	5	3.3
**Household monthly income (Naira, USD)**		
Not known	33	22.0
≤ 20,000 (**≤ **USD 44)	60	40.0
20,001—40,000 (44—88)	32	21.3
40,001 – 60,000 (90—132)	25	16.7
**Child**		
**Sex**		
Male	80	53.3
Female	70	46.7
**Age, months**		
0—5	49	32.7
6—11	32	21.3
12—17	38	25.3
18—23	31	20.7

The IRT parameter estimates for child feeding knowledge of teenage mothers in Abuja are presented in Table 2. Due to high difficulty and discrimination and lack of item fit, eight items were removed from the initial 31 items in this scale. The retained questions in this section (knowledge of food type, breastfeeding, and complementary feeding) generally exhibited good psychometric characteristics, and were approximate to, or within, the acceptable ranges of –3 to + 3 for difficulty, and 0.35 to 2.5 for discrimination. Difficulty levels ranged from – 2.473 for Q4 to 2.346 for Q11; 11 out of 23 items had values greater than 1, showing good difficulty. Discrimination index for all items ranged from 0.079 for Q21 to 2.568 for Q3. Three items (Q4, Q11 and 21) had lower discrimination levels than the recommended cut-off but were retained because their contents were important, and they met the criteria for other IRT parameters. All the items showed good fit (*p* > 0.05), except for Q10 and Q14, which had significant p-values. These were also retained because of their importance and good difficulty and discrimination values. Model fit was good, with an M2 value of 415.791, and CFI (0.911) and TLI (0.902) above the cut-off of 0.9. The RMSEA (0.073) was within the acceptable range of < 0.08. Marginal reliability was 0.813, indicating a high internal consistency.

**Table 2:** Parameter estimates of child feeding knowledge items in the Teen Moms Child Feeding Questionnaire for Sub-Saharan Africa

**Child feeding knowledge**	**Item parameters**		**Standardized chi-square fit index**		
	**Difficulty**	**Discrimination**	**Chi-square**	**Standardized loadings**	***P*-value**
**Food type**					
Q1. What is the first food for a newborn?	-1.290	2.426	5.836	0.958	0.35
Q3. What should infants < 6 months be fed?	-0.115	1.463	0.557	0.542	0.58
Q7. When a mother resumes work, what should her infant be fed?	0.673	1.037	0.666	0.332	0.37
**Exclusive breastfeeding**					
Q2. For how long should infants be fed with breast milk alone after birth?	-0.554	2.568	0.834	0.806	0.45
Q3. Why should infants be fed with breastmilk alone for some time after birth?	-0.697	2.403	1.772	0.843	0.22
Q4. How often should an infant < 6 months be breastfed daily?	-2.473	**0.299**	0.326	0.677	0.33
Q5. How can a mother keep up her breast milk supply?	1.685	0.649	0.321	0.251	0.53
Q6. What are the benefits of feeding an infant with breast milk only for mothers?	1.743	0.785	2.190	0.203	0.07
Q8. For how long can expressed breast milk keep without refrigeration?	1.439	1.356	0.883	0.124	0.66
Q9. What should a mother do to overcome difficulties with breastfeeding?	1.086	1.117	0.457	0.229	0.66
Q10. What are the effects of commercial/mixed feeding in infants < 6 months?	-1.196	0.403	2.587	0.618	0.03*
Q11. What are the signs that an infant needs to be breastfed?	2.346	**0.330**	0.088	0.684	0.62
Q12. For how long should an infant be suckled on one breast?	1.216	1.051	0.317	0.218	0.83
Q13. Why should an infant suckle on one breast for the stated length of time?	1.572	1.140	0.751	0.143	0.69
Q14. What are the signs that an infant is getting enough breast milk?	1.021	1.051	0.447	0.255	0.63
**Complementary feeding**					
Q14. At what age should complementary foods be introduced?	-2.088	0.440	3.178	0.715	0.03*
Q15. Why should complementary foods be introduced at the stated age?	-1.273	0.858	1.650	0.749	0.14
Q16. How many times daily should a 6-month-old receive complementary foods?	1.675	1.185	0.755	0.121	0.71
Q18. What quantity of food should an 8-month-old receive per meal?	-1.034	0.609	0.849	0.652	0.28
Q21. Should an infant receive thick or watery pap/porridges? Give reasons	-0.675	**0.079**	0.103	0.513	0.59
Q26. What should a mother do when her baby is vomiting, stooling and convulsing?	-1.772	0.721	0.259	0.782	0.63
Q27. How should you feed a baby who is refusing to eat complementary food?	1.161	0.815	0.578	0.280	0.39
Q28. What kind of diet provides the greatest amount of nutrients?	1.032	0.941	0.255	0.275	0.75

^*^Significant at *p* < 0·05, M_2_ = 415.791, Comparative Fit Index = 0.911, Tucker-Lewis Index = 0.902, Root Mean Square Error of approximation = 0.073

**Fig. 2:**
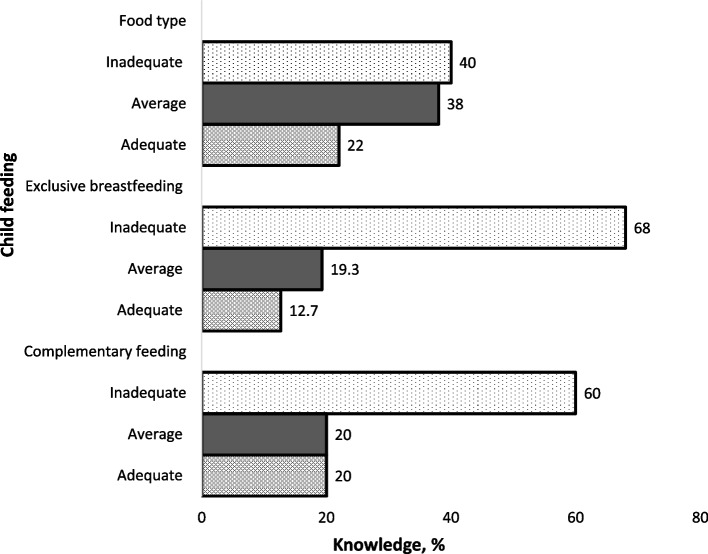
Summary of child feeding knowledge of teenage mothers in Abuja via the Teen Moms Child Feeding Questionnaire for Sub-Saharan Africa

Child feeding knowledge of teenage mothers in Abuja, assessed using the Teen Moms Child Feeding Questionnaire for sub-Saharan Africa is shown in Fig. 2. Inadequate knowledge of exclusive breastfeeding (68%), complementary feeding (60%) and infant food type (40%) were prevalent among study participants assessed using the Teen Moms Child Feeding Questionnaire for Sub-Saharan Africa.

Table 3 shows the confirmatory factor loadings of the attitude items in the Teen Moms Child Feeding Questionnaire for Sub-Saharan Africa. The model had a KMO value of 0.833, and a significant value (*p* < 0.05) for Bartlett’s test of sphericity; thus, meeting the criteria for factor analysis. Factor analysis with generalized least squares estimation and oblimin rotation produced a six-factor solution (i) exclusive breastfeeding; (ii) breast milk expression, (iii) meal frequency; (iv) responsive feeding; (v) dietary diversity; and (vi) barriers. All items in the attitude scale that had factor loadings greater than 0.3 were retained. After the elimination of six questions, the final attitude scale consisted of 17 items with factor loadings ranging from 0.301 to 0.937. Goodness-of-fit parameters met the recommended criteria. Although the chi-square test was significant, the normed chi-square value was 1.8, which is considered a good fit. Comparative Fit (0.934) and Tucker Lewis (0.914) indices were greater than 0.9, showing good model fit. The root mean square error of approximation was 0.072 and the standardized root mean square residual was 0.068; all were below the suggested cut-off of 0.08.

**Table 3:** Confirmatory factor loadings of the attitude items in the Teen Moms Child Feeding Questionnaire for Sub-Saharan Africa

Attitude questions	Confirmatory factor loadings					
	**Exclusive breastfeeding**	**Breast milk expression**	**Meal frequency**	**Responsive feeding**	**Dietary diversity**	**Barriers**
Q2. Exclusive breastfeeding does not allow the baby grow well	0.642*					
Q3. It is difficult for me to breastfeed exclusively for 6 months	0.926*					
Q4 I am confident I can successfully breastfeed exclusively for 6 months	0.922*					
Q6. It is a dirty and unsafe practice to express breast milk for my child		0.825*				
Q7. It is difficult for me to express breast milk for my child		0.850*				
Q8. I am confident I can successfully express breast milk for my child		0.937*				
Q10. It is too much to feed infants five times daily at 12 months			0.790*			
Q11. It is difficult to feed my child five times daily at 12 months			0.737*			
Q12. I am confident I can feed my child five times daily at 12 months			0.930*			
Q13. If my child is refusing to eat, it is better to force the child to eat				0.632*		
Q15. It is difficult to feed my child responsively				0.640*		
Q16. I am confident I can successfully feed my child responsively				0.787		
Q17. It is good to include four food groups in my child’s meals each day					0.301*	
Q19. It is difficult to include four food groups in my child’s meals each day					0.870*	
Q20. I am confident I can include four food groups in my child’s meals each day					0.863*	
Q21. I am having difficulties in feeding my child well because I am young						0.614*
Q22. I want to feed my child well but do not have support from family & friends						0.723*

^*^Items with *p* < 0·05, chi-square test statistic = 184.904, degrees of freedom = 104, chi-square *p*-value < 0.05, normed chi-square = 1.8, Comparative Fit Index = 0.934, Tucker-Lewis Index = 0.914, Root Mean Square Error of approximation = 0.072, Standardized Root Mean Square Residual = 0.070

**Fig. 3:**
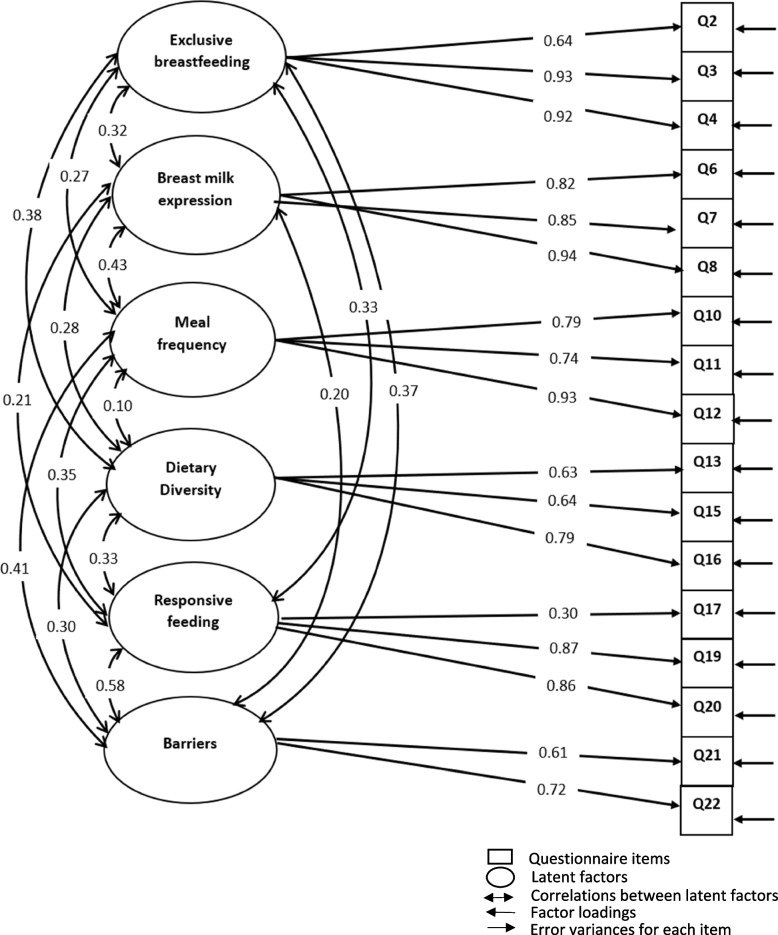
Confirmatory path analysis of the attitude scale of the Teen Moms Child Feeding Questionnaire for Sub-Saharan Africa

Figure 3 shows the confirmatory path analysis of the attitude scale of the Teen Moms Child Feeding Questionnaire for Sub-Saharan Africa. This depicts the relationship between the latent and observed variables. Circles represent the latent factors, double-headed arrows show the unique correlations between the latent factors, and square boxes indicate the questionnaire items (observed/measured variables). The factor loadings of each item are depicted by single arrows pointing from latent to observed variables, showing the effects of each factor on the observed variable. The single arrows pointing from the right end towards the observed variables indicate the measurement error variance specific to each of the 16 questions. From the path analysis, it is evident that all six factors are correlated, with coefficients ranging from 0.01 to 0.58. There are no substantial cross-loadings of items on more than one factor, since each questionnaire item loaded on only one latent factor.

Reliability of the attitude section of the Teen Moms Child Feeding Questionnaire for sub-Saharan Africa is presented in Table 4. Cronbach’s alpha for the overall attitude scale was 0.843, showing a high internal consistency. Cronbach’s alpha for all the attitude sub-scales were within the acceptable range of 0.6 or greater. Item-total correlations for all items ranged from 0.33 to 0.64, above the suggested minimum value of 0.30. The values of Cronbach’s alpha if item deleted were all low (0.25 – 0.58) for each item, and lower than the Cronbach’s alpha level for the complete scale; this further confirms the reliability of the scale. The intraclass correlation coefficients of the subscales were greater than 0.75, showing excellent test–retest reliability.

**Table 4:** Internal consistency and test–retest reliability of the attitude section of the Teen Moms Child Feeding Questionnaire for Sub-Saharan Africa

Attitude subscale	Cronbach’s alpha	Test–retest reliability		Items	Mean (SE)	Item-total correlation^a^	Alpha if item deleted
		**Intraclass correlation coefficient**	**Confidence intervals**				
Exclusive breastfeeding	0.86	0.78*	0.62, 0.88	Q2	4.1 (0.020)	0.48	0.43
				Q3	3.8 (0.020)	0.62	0.53
				Q4	4.0 (0.020)	0.63	0.54
Breast milk expression	0.60	0.76*	0.59, 0.87	Q6	3.2 (0.021)	0.61	0.56
				Q7	3.0 (0.020)	0.55	0.50
				Q8	3.1 (0.021)	0.63	0.58
Meal frequency	0.86	0.90*	0.81, 0.95	Q10	3.8 (0.020)	0.49	0.43
				Q11	3.6 (0.020)	0.50	0.46
				Q12	3.8 (0.021)	0.64	0.57
Responsive feeding	0.71	0.91*	0.83, 0.95	Q13	3.5 (0.020)	0.48	0.43
				Q15	3.8 (0.019)	0.33	0.25
				Q16	4.1 (0.019)	0.42	0.34
Dietary diversity	0.86	0.81*	0.67, 0.90	Q17	3.5 (0.020)	0.55	0.47
				Q19			
				Q20	3.7 (0.020)	0.54	0.46
Barriers	0.61	0.97*	0.95, 0.99	Q21	3.2 (0.019)	0.40	0.35
				Q22	3.8 (0.019)	0.46	0.40
Complete scale	0.84	0.89*	0.80, 0.94				

^a^Corrected item-total correlation

*p < 0.05

**Fig. 4:**
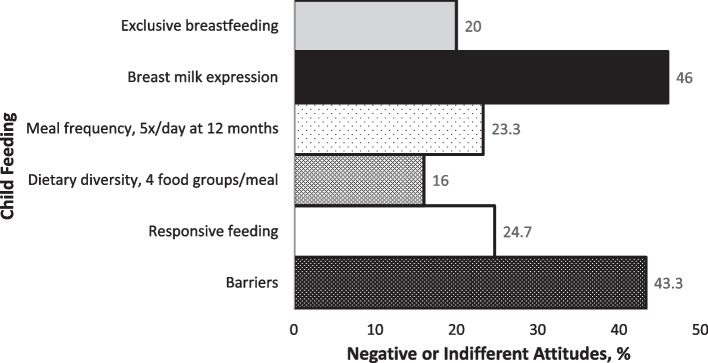
Summary of child feeding attitudes of teenage mothers in Abuja via the Teen Moms Child Feeding Questionnaire for Sub-Saharan Africa

Figure 4 presents a summary of child feeding attitudes of teenage mothers in Abuja, evaluated using the Teen Moms Child Feeding Questionnaire for sub-Saharan Africa. Almost half (46%) of the mothers assessed via the Teen Moms Child Feeding Questionnaire for Sub-Saharan Africa had either negative or indifferent attitudes towards expression of breast milk. Similarly, large proportions (43.3%) of the mothers reported barriers in feeding their children either due to age, or inadequate support from family and friends.

## Discussion

The aim of the present study was to evaluate the psychometric properties of an instrument to measure knowledge and attitudes of teenage mothers towards infant and young child feeding. The study established content and construct validity, as well as reliability, of the Teen Moms Child Feeding Questionnaire for Sub-Saharan Africa. The psychometric properties of the Teen Moms Child Feeding Questionnaire for Sub-Saharan Africa were generally adequate. The knowledge scale in the current study was unidimensional, yet, it covered a wide range of issues concerning child feeding, including knowledge of food type, exclusive breastfeeding and complementary feeding. The use of item response theory in the knowledge domain ensured that questions retained were mostly of adequate difficulty, and discriminated well between participants who knew the concepts and those that did not. Adequate model fitness and marginal reliability were established for this scale. The retained questions in the attitude scale of the questionnaire loaded onto six constructs (exclusive breastfeeding, breast milk expression, meal frequency, responsive feeding, dietary diversity and barriers) with acceptable factor loadings, and good model fit and reliability indices. These confirmed constructs are essential for the success of infant and young child feeding, as they provide relevant information on specific attitudes that will need to be addressed in behavior change communications.

In the current study, the IRT parameters, model fit indices and measures of reliability of both the knowledge and attitude domains met the established criteria. Difficulty (– 2.473 to 2.346) and discrimination (0.079 to 2.568) indices of the knowledge scale covered a wide range of ability levels. This is in contrast with the findings of Das et al. (2020), of difficulty and discrimination levels of 0.33 – 0.87 and 0.12 – 0.44, respectively, in a validation study of a nutrition knowledge questionnaire for parents of young children in India [84]. Nonetheless, the findings of the present study are similar to the range of values obtained by Zakria et al. (2019) of –5.6 to + 1.7 and 0.3 – 2.4, respectively, in the validation of a Child Feeding Knowledge, Attitudes and Practice Questionnaire for caregivers in Malaysia [49].

In the present study, the factor analysis retained questions with a wide range of factor loadings (0.301—0.937). This is consistent with values of 0.422—0.860 reported by Liu et al. (2023) in the validation of a responsive feeding questionnaire among caregivers of Chinese toddlers [85]. Similarly, in a Brazilian study to adapt and validate the Infant Feeding Style Questionnaire, factor loadings were shown to range from 0.30—0.89 [86]. Also, Davie et al. (2021) showed loadings of 0.40—0.93 on validation of the Beliefs About Breastfeeding Questionnaire in the United Kingdom [87]. Nonetheless, values from the validation of the Maternal Distraction Questionnaire in the United States, and the Feeding Practices and Structure Questionnaire by Jansen et al. (2021) in Australia showed relatively narrower ranges of factor loadings (0.40 – 0.69 and 0.58 – 0.92, respectively) [88, 89].

Intraclass correlation coefficients (> 0.75) in this study were similar to the values obtained by Liu et al, 2023 [85], but higher than the findings of 0.615 by Oliveira et al. (2020), in a study to validate a questionnaire for assessment of knowledge on complemetary feeding in Brazil [90]. Cronbach’s alphas (0.60 – 0.86) in the current study ranged more broadly in comparison with the values 0.81 – 0.87 and 0.79 – 0.86 shown by Liu et al. (2023) and Ventura et al. (2020), respectively [85, 88]. Nonetheless, Cronbach’s alpha values in the present study were lower than the findings of 0. 70 – 0.92 in Australia, yet higher than those reported by Pedroso et al. (0.42 to 0.75) [89, 90].

The CFI and TLI for both sections of the current questionnaire were above 0.9 and slightly higher than those reported by Zakria et al. (0.79 and 0.80, respectively), but in agreement with values > 0.9 reported by Jansen et al. (2021), Pedroso et al. (2021), and by Purwaningrum et al. (2018) on validation of a parental child feeding questionnaire in Indonesia [49, 86, 89, 91]. Additionally, the normed chi-square value of 1.8 in the current study was slightly higher than the value 1.3 obtained by Purwaningrum et al. (2018) [91]. Consistent with the findings of Zakria et al. (2019), Purwaningrum et al. (2018) and Jansen et al. (2021), the RMSEA and SRMR for both scales in the present study were acceptable, and marginal reliability was high [49, 89, 91]. In sum, these values reflect a valid and reliable scale. Additionally, these results are generalizable to the rural areas of Nigeria and similar contexts in sub-Saharan Africa; however, the specific findings may differ across various settings.

The Teen Moms Child Feeding Questionnaire for sub-Saharan Africa is different from existing tools in many ways. It measures both knowledge and attitudes concerning breastfeeding and complementary feeding, and is validated in the population it is intended for (adoelsecnt mothers). Additionally, it evaluates individual constraints (being young), and interpersonal barriers (lack of support from family and friends), that may negatively affect IYCF. Instruments utillized by Ikobah et al. (2020), Pillay et al. (2018), Odukoya et al. (2022) and Leshi et al (2016 and 2022) focused solely on breastfeeding [28–33]. A survey by Samuel et al. (2016) evaluated knowledge of both breastfeeding and complementary feeding in a different population (health workers) [29]. Conti et al. validated a general scale for nutrition and food knowledge among women [34]. Thus, the current, newly developed questionnaire is a comprehensive instrument that may be useful for identifying previously overlooked gaps in knowledge and attitudinal barriers to IYCF, in low-income, adolescent mothers.

The validated instrument in this study was designed to empirically gauge the level of awareness and disposition of teenage mothers towards child feeding. To reduce potential participant biases due to acquiescence (responding in the affirmative to questions regardless of content) and social desirability (providing answers perceived as desirable), the knowledge scale was presented in a semi-structured format. Thus, the scale had both closed- and open-ended questions, worded to be neutral, concise, and non-leading [92, 93]. This is important, as the promotion of exclusive breastfeeding as a moral duty by health workers has been correlated with feelings of guilt, shame and condemnation among mothers [94]. Thus, the use of questions like “should infants be breastfed exclusively?” were avoided to reduce the likelihood of participants responding solely based on either of the biases.

Strengths of this present validated scale include ease of administration in rural low-income, teenage mothers with limited education. One limitation of the current study is the inclusion of a large number of adolescent mothers > 17 years. This is because the current questionnaire validation was part of a larger study, for which few younger teenage mothers in the study area met the inclusion criteria. Also, translation from English to Hausa and back to English could be a limitation. Nonetheless, the research team consisted of Hausa-speaking individuals.

## Conclusion

The Teen Moms Child Feeding Questionnaire for Sub-Saharan Africa was developed and found to be a valid tool for assessing the knowledge and attitudes of teenage mothers towards infant and young child feeding in rural Nigeria. This questionnaire will be a useful instrument for nutrition professionals, community health workers, non-profit organizations, researchers in child health promotion and other stakeholders to evaluate maternal child feeding. This information can be utilized in future interventions to ameliorate child undernutrition, particularly among children born to teenage mothers.

### Supplementary Information

**Additional file 1.**

## Data Availability

The datasets generated and/or analysed during the current study are not publicly available yet, because this study is part of a larger, ongoing research project. Nonetheless, the data are available from the corresponding author on reasonable request.

## Abbreviations

IYCF: Infant and Young Child Feeding
IRT: Item Response Theory
CFA: Confirmatory Factor Analysis
WHO: World Health Organization
UNICEF: United Nations Children’s Fund
*S-X2*: Standardized chi-square
KMO: Kaiser–Meyer–Olkin
EFA: Exploratory Factor analysis
TLI: Tucker Lewis Index
CFI: Comparative Fit Index
RMSEA: Root Mean Square Error of Approximation
SRMR: Standardized Root Mean Square Residual
