# Supplementary material for: Development and validation of the Teen Moms Child Feeding Questionnaire for Sub-Saharan Africa
Source: BMC Public Health. 2023 Aug 4;23:1487. doi: 10.1186/s12889-023-16365-5 (PMC10401754; doi:10.1186/s12889-023-16365-5)
Supplement: Supplementary file 1 — Additional file 1. [file 12889_2023_16365_MOESM1_ESM.docx]

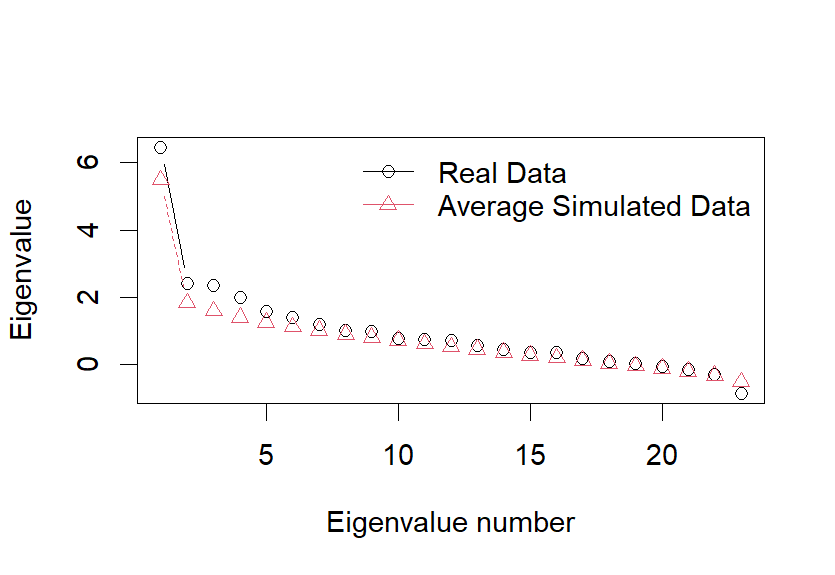


**Online supplementary material 1. Eigenvalue plot showing the modified parallel analysis to establish unidimensionality**

Modified parallel analysis involves the comparison of a scree plot constructed from the eigenvalues of a simulated data set with a scree plot from the actual observed data (a). The scale is unidimensional when there is only one eigenvalue above the point where the lines of the two scree plots intersect, or when there is no substantial difference in the magnitude of the second eigenvalue of the actual data and the second eigenvalue of the simulated data (modified parallel analysis plot in the supplementary material) (b, c).

a. Kanyongo GY. The influence of reliability on four rules for determining the number of components to retain. J Mod Appl Stat Methods. 2005;5(2):7.

b. Drasgow, F.; Lissak, R.I. Modified parallel analysis: A procedure for examining the latent dimensionality of dichotomously scored item responses. J Appl Psychol. 1983;68, 363–373.

c. Carol M. Woods, Michael C. Edwards, Factor Analysis and Related Methods. Editor(s): C.R. Rao, J.P. Miller, D.C. Rao, Essential Statistical Methods for Medical Statistics, North-Holland, 2011, Pages 174-201.
